# Independent prognostic genes and mechanism investigation for colon cancer

**DOI:** 10.1186/s40659-018-0158-7

**Published:** 2018-04-13

**Authors:** Chunsheng Li, Zhen Shen, Yangyang Zhou, Wei Yu

**Affiliations:** 1 Gastrointestinal Colorectal and Anal surgery, China-Japan Union Hospital of Jilin University, No. 126 Xiantai Street, Changchun, Jilin 130033 China; 2 Department of Neurology, The First Hospital of Jilin University, Changchun, Jilin 130021 China

**Keywords:** Colon cancer, Differentially expressed microRNAs, Function and pathway analysis, Independent prognostic gene, Overall survival, Disease-free survival

## Abstract

**Propose:**

We aimed to explore the potential molecular mechanism and independent prognostic genes for colon cancer (CC).

**Methods:**

Microarray datasets GSE17536 and GSE39582 were downloaded from Gene Expression Omnibus. Meanwhile, the whole CC-related dataset were downloaded from The Cancer Genome Atlas (TCGA) database. Differentially expressed mRNA (DEMs) were identified between cancer tissue samples and para-carcinoma tissue samples in TCGA dataset, followed by the KEGG pathway and GO function analyses. Furthermore, the clinical prognostic analysis including overall survival (OS) and disease-free survival (DFS) were performed in all three datasets.

**Results:**

A total of 633 up- and 321 down-regulated mRNAs were revealed in TCGA dataset. The up-regulated mRNAs were mainly assembled in functions including extracellular matrix and pathways including Wnt signaling. The down-regulated mRNAs were mainly assembled in functions like Digestion and pathways like Drug metabolism. Furthermore, up-regulation of UL16-binding protein 2 (*ULBP2*) was associated with OS in CC patients. A total of 12 DEMs including Surfactant Associated 2 (*SFTA2*) were potential DFS prognostic genes in CC patients. Meanwhile, the *GRP* and Transmembrane Protein 37 (*TMEM37*) were two outstanding independent DFS prognostic genes in CC.

**Conclusions:**

*ULBP2* might be a potential novel OS prognostic biomarker in CC, while *GRP* and *TMEM37* could be served as the independent DFS prognostic genes in CC. Furthermore, functions including extracellular matrix and digestion, as well as pathways including Wnt signaling and drug metabolism might play important roles in the process of CC.

## Background

Colon cancer (CC) is one of the best-understood neoplasms from a genetic perspective [1]. Globally, CC is the third most common type of cancer making up about 10% of all cases [2]. There are over 1.4 million new cases and 694,000 deaths from the CC in 1 year worldwide [3]. Treatments for CC may include some combination of surgery, radiation therapy, chemotherapy and targeted therapy [4, 5]. Although the integrated surgical strategies increased the survival rate, the removal of the colon may not suffice as a preventative measure because of the high risk of rectal cancer if the rectum remains [6].

Numerous data indicate that the aberrant accumulation of genetic changes functions as vital roles in initiation and development of colon and rectal cancer [7, 8]. Messenger RNA (mRNAs) are important regulatory molecules which can affect a variety of cellular and molecular targets in various cancers including CC [9]. A previous study shows that different types of mRNA can be used as tissue- and exosome-based diagnostic biomarkers for human CC [10]. The high expression of mRNA such as interleukin-6 can be used as a predictor of relapse in CC [11]. Furthermore, certain kinds of mRNA can be used to predict survival in CC patients [12]. A previous study shows that Ephrin-A1 mRNA is associated with poor prognosis of CC patients based on short disease-free survival (DFS) data [13]. Alexopoulou et al. showed that Kallikrein Related Peptidase 11 mRNA expression could predict poor DSF and overall survival (OS) in colorectal adenocarcinoma patients [14]. Thus, a better knowledge of the molecular mechanisms and cancer associated gene is vital for the early diagnosis and personalized care of CC patients. However, the independent prognostic gene associated with death and recurrence of CC is still unclear.

In previous studies, Smith et al. [15, 16] and Marisa et al. [17] tried to predict the mRNAs associated with the recurrence and death in CC patients based on gene expression profiles. Although some valuable biological markers for prognosis of CC has been revealed, the limited sample size and survival evaluation in these studies are not benefit for the investigation of independent prognostic genes. Based on the previous results from Smith et al. and Marisa et al. The Cancer Genome Atlas (TCGA) dataset associated with CC were added in the current bioinformatics study. By comparing the data between cancer tissue samples and para-carcinoma tissue samples in TCGA dataset, the differentially expressed mRNAs (DEMs) investigation, functional and pathway enrichment analysis were performed. Furthermore, prognostic analysis including overall survival and disease-free survival rate investigation was performed based on data in all three datasets. We hoped to explore molecular mechanism of CC, and identify candidate independent prognostic genes for CC prognosis.

## Methods

### The mRNA microarray data

Microarray dataset GSE17536 [15, 16] and GSE39582 [17] were downloaded from Gene Expression Omnibus (GEO, http://www.ncbi.nlm.nih.gov/geo/) database. A total of 177 invasive murine CC cells sample were included in dataset GSE17536. Meanwhile, a total of 566 CC samples were included in GSE39582. The gene expression profile data of GSE17536 and GSE39582 were all generated based on the platform of Affymetrix Human Genome U133 Plus 2.0 Array (GPL570 [HG-U133_Plus_2]). Furthermore, the whole CC-related dataset in TCGA database (https://cancergenome.nih.gov/) were downloaded.

### Data preprocessing and DEMs identification

Normalized RNA-seq data (including 24,991 genes) of TCGA dataset were downloaded for the further investigation. Meanwhile, the CEL source files of GSE17536 and GSE39582 were processed into background adjustment [18], quantile normalization [19], summarization [20] and Log2 fold change [21] using Robust Multi-array Average (RMA) algorithm [22] in Affy software [23]. Finally, a total of 22,844 and 22,854 genes were obtained from GSE39582 and GSE17536 respectively after data processing.

The DEMs were identified between cancer tissue samples and para-carcinoma tissue samples in TCGA dataset based on Student’s t test [24]. P value < 0.05 and fold change > 2 (or < 1/2) were defined to be statistically significant.

### Functional annotation and pathway enrichment analysis of DEMs

The Database for Annotation, Visualization and Integrated Discovery (DAVID) [25] is a gene functional classification tool that provides a comprehensive set of functional annotation tools for investigators to understand biological meaning behind large list of genes. By DAVID software, the Gene Ontology (GO, http://www.geneontology.org) functional annotation [26] and Kyoto Encyclopedia of Genes and Genomes (KEGG) (http://www.genome.jp/kegg/pathway.html) [27] pathway analyses were performed on DEMs in TCGA dataset. P < 0.05 was chosen as the cut-off criterion for the enrichment analysis. The results of GO function and KEGG pathway analysis were visualized by ErichmentMap [28] software.

### Clinical prognostic analysis

Based on the DEMs obtained in TCGA dataset, the clinical prognostic analyses including overall survival (OS) and disease-free survival (DFS) analysis were performed on the datasets which have clinical prognostic information. All the three datasets including TCGA, GSE17536 and GSE39582 were used for the overall survival analysis in the present study. Meanwhile, two datasets including GSE17536 and GSE39582 were used for the disease-free survival analysis. The mRNAs in all datasets were divided into high expression group (H group) and low expression group (L group) according to the mean value of DEMs. The survival estimation and survival curve examination were performed using Kaplan–Meier method [29] and log-rank test [30], respectively. The hazard ratio (HR) was estimated with the single variable Cox proportional risk regression model [31]. The independent analysis of prognostic factor was performed based on multivariable Cox proportional risk regression model [32]. Based on the Cox proportional risk regression model, the outstanding DEMs of the single variable in each dataset were considered as the corrected variables. P < 0.05 was considered statistically significant.

## Results

### DEMs investigation

**Fig. 1:**
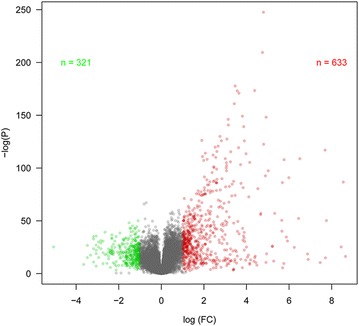
Volcano plot showing differentially expressed mRNAs. The X-axis represents the log fold-change (FC) values; the Y-axis represents the—log P values; green point represents the down-regulated mRNAs; the red point represents the up-regulated mRNAs; those points having a FC larger than 2 (or FC < 1/2) are shown in gray

With P value < 0.05 and fold change > 2 (or < 1/2), a total of 954 DEMs including 633 up- and 321 down-regulated mRNA were revealed in TCGA dataset (Fig. 1).

### Function annotation and pathway enrichment investigation

**Fig. 2:**
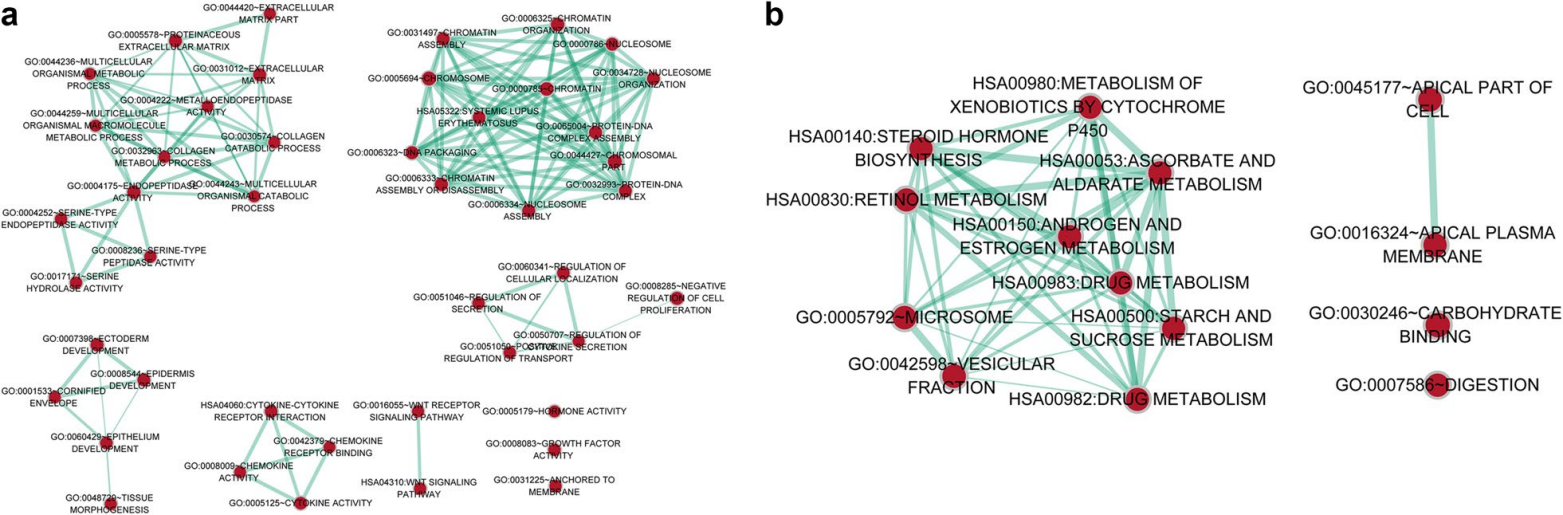
The GO function and KEGG pathways enrichment analysis for the up- and down-regulated mRNAs. **a** the network constructed by the up-regulated mRNAs; **b** the network constructed by the down-regulated mRNAs; GO represents Gene Ontology; KEGG represents Kyoto Encyclopedia of Genes and Genomes; the red point represents a certain name of GO function or KEGG pathway; the green line represents the interaction between two points; the thickness of the line indicates the number of overlapping genes between the different gene sets

With P < 0.05, the GO function and KEGG pathway of DEMs were investigated, followed by visualized using ErichmentMap software. As showed in Fig. 2a, extracellular matrix (GO:0031012), chromatin assembly or disassembly (GO:0006333) and endopeptidase activity (GO:0004175) were outstanding functions assembled with up-regulated DEMs. Meanwhile, the Systemic lupus erythematosus (hsa05322) and Wnt signaling pathway (hsa04310) were two outstanding pathways enriched by up-regulated DEMs (Fig. 2a).

As showed in Fig. 2b, Microsome (GO:0005792), Vesicular fraction (GO:0042598) and Digestion (GO: 0007586) were outstanding functions assembled with the down-regulated DEMs, while the Drug metabolism (hsa00983), Androgen and estrogen metabolism (hsa00150, Genes) and Retinol metabolism (hsa00830) were outstanding pathways enriched by down-regulated DMEs (Fig. 2b).

### Investigation of CC associated OS prognostic genes

**Fig. 3:**
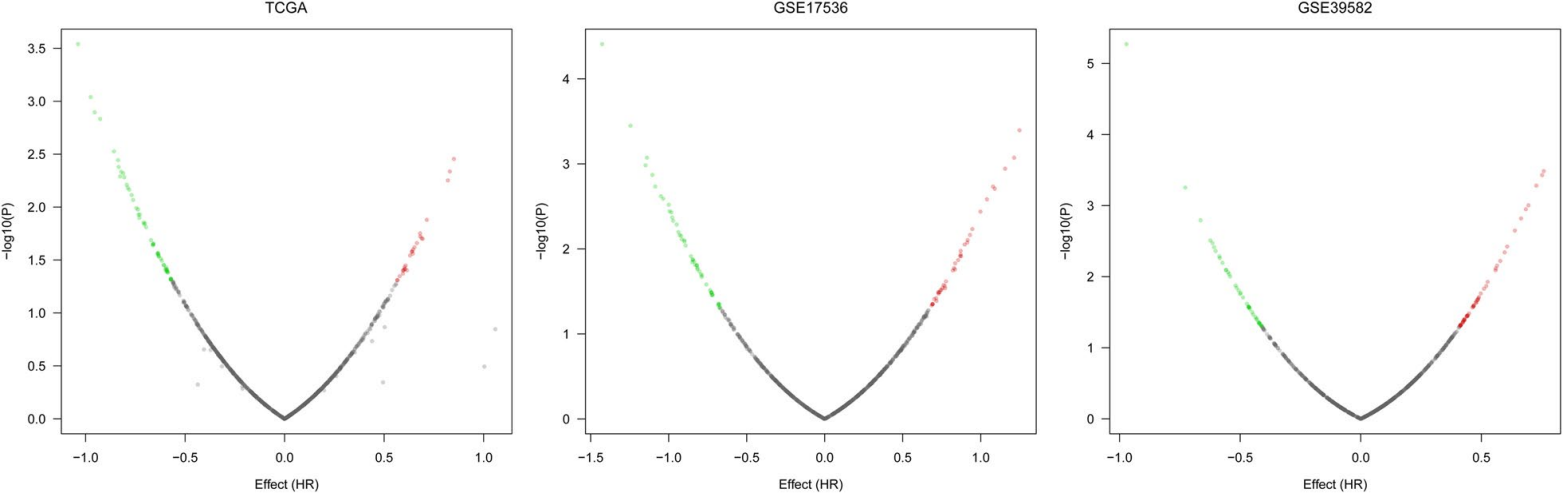
Cox proportional risk regression plot analyses between differentially expressed microRNAs and overall survival in colon cancer. X-axis represents the logarithm of hazard ratio (HR), the positive expression is associated with better prognosis, and negative expression of gene expression is associated with poor prognosis; Y-axis represents logarithm of P value for the log-rank test

**Fig. 4:**
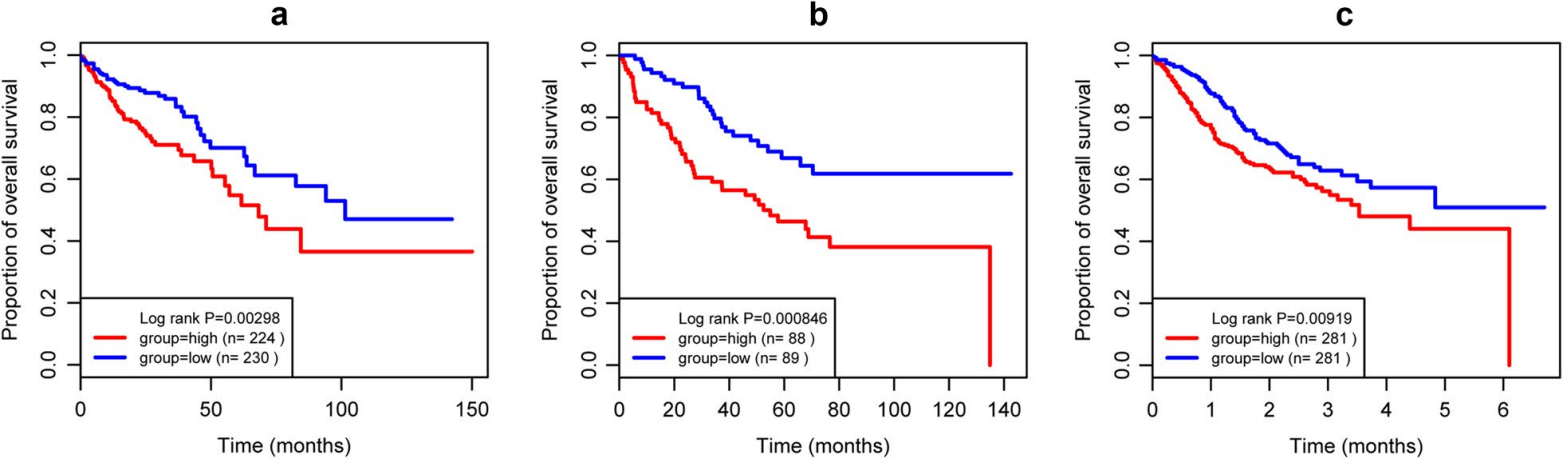
Kaplan-Meier analyses for the relation between shorter overall survival and UL16 Binding Protein 2 gene in colon cancer. **a** The TCGA dataset; **b** the GSE17536 dataset; **c** the GSE39582 dataset

The relationship between DMEs and OS in each dataset was showed in Fig. 3. The results showed that UL16-binding protein 2 (*ULBP2*) was the outstanding gene in all three dataset including TCGA [Log-rank P 0.0030, HR: 0.552, 95% confidence interval (CI) 0.37–0.82], GSE17536 (Log-rank P 0.0005, HR: 0.380, 95% CI 0.22–0.67) and GSE39582 (Log-rank P 0.0092, HR: 0.685, 95% CI 0.51–0.91). Furthermore, up-regulation of *ULBP2* gene was associated with shorter OS of CC patients. The detail information was showed in Fig. 4.

### Investigation of DFS prognostic gene

**Fig. 5:**
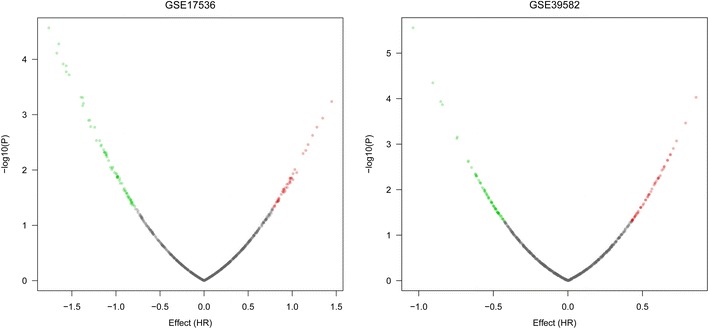
Cox proportional risk regression plot analyses between differentially expressed microRNAs and disease-free survival in colon cancer. X-axis represents the logarithm of hazard ratio (HR), the positive expression is associated with better prognosis, and negative expression of gene expression is associated with poor prognosis; Y-axis represents logarithm of P value for the log-rank test

**Fig. 6:**
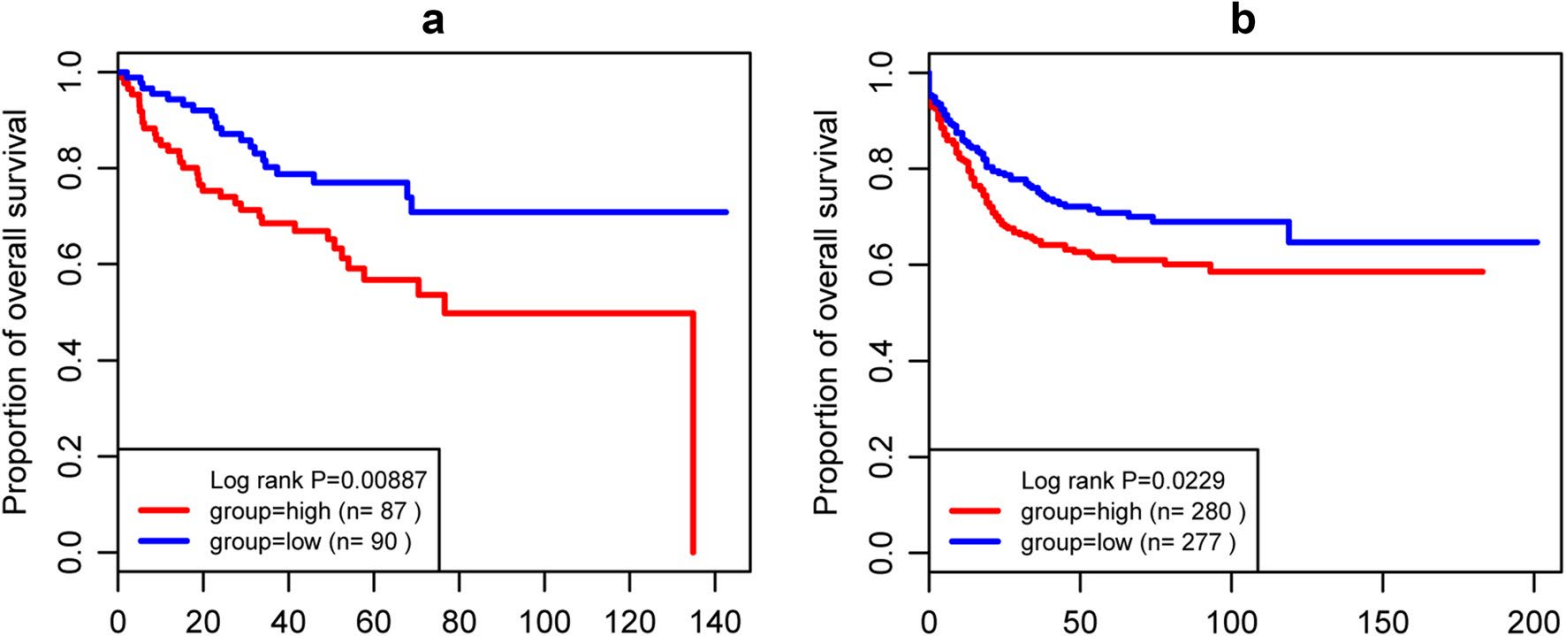
Kaplan-Meier analyses for the relation between shorter disease-free survival and Surfactant Associated 2 gene in colon cancer. **a** The GSE17536 dataset; **b** the GSE39582 dataset

The relationship between DMEs and DFS prognostic genes associated with CC in each dataset was showed in Fig. 5. The results showed that a total of 12 DEMs were outstanding in both GSE17536 and GSE39582, including Surfactant Associated 2 (*SFTA2*), LEM Domain Containing 1 (*LEMD1*), Cartilage Oligomeric Matrix Protein (*COMP*), Kinesin Family Member 26B (*KIF26B*), Kallikrein Related Peptidase 10 (*KLK10*), Matrix Metallopeptidase 11 (*MMP11*), Gastrin Releasing Peptide (*GRP*), Twist Family BHLH Transcription Factor 1 (*TWIST1*), Regulator Of G Protein Signaling 16 (*RGS16*), Collagen Type VIII Alpha 1 Chain (*COL8A1*), Transmembrane Protein 37 (*TMEM37*), Rho GTPase Activating Protein 44 (*ARHGAP44*) (Table 1). Notably, the relation between up-regulation of *SFTA2* and short DFS in CC patients was showed in Fig. 6.

**Table 1:** The outstanding disease-free prognostic genes for colon cancer

Gene	FC	P	GSE17536			GSE39582		
			Log-rank P	HR	95% CI	Log-rank P	HR	95% CI
SFTA2	63.45	1.6E−91	8.87E−03	0.488	0.49 (0.28–0.84)	2.29E−02	0.708	0.71 (0.53–0.95)
LEMD1	32.47	4.3E−98	1.32E−02	0.506	0.51 (0.29–0.88)	1.47E−02	0.692	0.69 (0.51–0.93)
COMP	14.33	4.3E−82	3.70E−03	0.444	0.44 (0.25–0.78)	1.48E−02	0.691	0.69 (0.51–0.93)
KIF26B	6.86	2.4E−64	5.85E−03	0.466	0.47 (0.27–0.81)	1.09E−02	0.679	0.68 (0.5–0.92)
KLK10	6.06	1.1E−99	5.28E−03	0.462	0.46 (0.26–0.81)	7.06E−03	0.666	0.67 (0.49–0.9)
MMP11	3.74	6.8E−127	2.96E−03	0.44	0.44 (0.25–0.77)	3.68E−02	0.731	0.73 (0.54–0.98)
GRP	3.22	2.4E−15	1.43E−02	0.515	0.52 (0.3–0.88)	1.16E−04	0.554	0.55 (0.41–0.75)
TWIST1	2.59	1.3E−28	4.07E−02	0.562	0.56 (0.32–0.98)	4.29E−02	0.736	0.74 (0.55–0.99)
RGS16	2.29	2.1E−25	1.90E−04	0.346	0.35 (0.19–0.62)	3.48E−02	0.726	0.73 (0.54–0.98)
COL8A1	2.01	9.6E−15	1.68E−04	0.338	0.34 (0.19–0.61)	6.21E−03	0.661	0.66 (0.49–0.89)
TMEM37	0.47	1.3E−23	1.41E−02	1.986	1.99 (1.14–3.47)	1.28E−02	1.457	1.46 (1.08–1.96)
ARHGAP44	0.45	4.8E−20	4.88E−02	1.717	1.72 (1–2.96)	4.58E−02	1.349	1.35 (1–1.82)

*FC* fold change, *HR* hazard ratio, *CI* confidence interval

P < 0.05 was considered as significant different

### Independent DFS prognostic genes analysis

The independent DFS prognostic genes in CC were explored in GSE17536 and GSE39582. The results showed that *GRP* and *TMEM37* were outstanding in totally 12 DEMs after the multivariate Cox proportional risk regression (Table 2).

**Table 2:** The potential disease-free survival independent prognostic gene for colon cancer

Gene	GSE17536			GSE39582		
	P	HR	HRse	P	HR	HRse
SFTA2	0.07	0.542	0.28–1.051	0.114	0.775	0.565–1.063
LEMD1	0.465	0.789	0.417–1.491	0.156	0.796	0.581–1.091
COMP	0.871	0.943	0.466–1.91	0.953	0.988	0.658–1.483
KIF26B	0.915	1.042	0.491–2.211	0.808	0.955	0.656–1.39
KLK10	0.319	0.705	0.355–1.401	0.364	0.861	0.623–1.19
MMP11	0.566	0.807	0.388–1.679	0.498	1.148	0.77–1.711
GRP	0.275	0.707	0.38–1.317	0.011	0.612	0.418–0.895
TWIST1	0.215	1.706	0.734–3.967	0.932	1.017	0.686–1.509
RGS16	0.184	0.613	0.298–1.262	0.917	0.982	0.696–1.385
COL8A1	0.088	0.445	0.175–1.128	0.467	0.856	0.563–1.301
TMEM37	0.012	2.177	1.183–4.007	0.114	1.289	0.941–1.766
ARHGAP44	0.931	0.973	0.524–1.807	0.642	1.08	0.782–1.49

*HR* hazard ratio, *HRse* hazard ratio standard error

P < 0.05 was considered as significant different

## Conclusion

In sum, *ULBP2* might be a potential novel OS prognostic biomarker in CC, while *GRP* and *TMEM37* could be served as the independent DFS prognostic genes in CC. Furthermore, functions including extracellular matrix and digestion, as well as pathways including Wnt signaling and drug metabolism may play important roles in the process of CC.

## Discussion

The current informatics study revealed the potential independent prognostic genes for survival of CC, as well as the molecular mechanism during CC process. A total of 633 up- and 321 down-regulated DEMs were revealed between cancer tissue samples and para-carcinoma tissue samples. The up-regulated mRNAs were mainly assembled in functions including Extracellular matrix and pathways including Wnt signaling. The down-regulated mRNAs were mainly assembled in functions like Digestion and pathways like Drug metabolism. Furthermore, up-regulation of *ULBP2* was associated with OS in CC patients. A total of 12 genes including *SFTA2* were potential DFS prognostic genes in CC patients. Meanwhile, the *GRP* and *TMEM37* were two outstanding independent DFS prognostic genes in CC.

The extracellular matrix is a collection of extracellular molecules secreted by cells that provides structural and biochemical support to the surrounding cells [33]. Extracellular matrix plays an important role in cancer progression as a dynamic niche [34]. A previous study shows that matrix metalloproteinase-9 is an important marker for analysis of the postoperative prognosis and risk of metastases in patients with colorectal cancer [35]. Recently, Wang et al. showed that the extracellular matrix protein mindin attenuated colon cancer progression by blocking angiogenesis via Egr-1-mediated regulation [36]. Despite of extracellular matrix, the digestion function is closed related to cancer preventative activity [37]. The microflora in digestion system degrades a wide variety of organic compounds including food additives, drugs, bile salts and cholesterol which may be relevant to the development of CC [38]. A previous study shows that peptides derived from in vitro gastrointestinal digestion can inhibit human colon cancer cells proliferation and inflammation [39]. In the present study, GO function analysis showed that the up- and down-regulated mRNAs were mainly assembled in extracellular matrix and digestion. Thus, our results reveal that the abnormal of extracellular matrix and digestion function may play vital roles in the progression of CC.

Furthermore, the clinical importance of Wnt signaling pathway has been demonstrated in various diseases including colorectal cancer [40]. A previous study shows that alteration in the Wnt signaling pathway is frequently observed in colorectal cancer with microsatellite instability [41]. Inhibiting the Wnt signaling pathway may be a fruitful strategy for targeting chemotherapy-resistant CC cells [42]. Moreover, drug metabolism is the metabolic breakdown of drugs by living organisms [43]. Landmann indicated that drug metabolism determined resistance of colorectal cancer to resorcinol-based heat shock protein 90 inhibitors [44]. In the present study, Wnt signaling and drug metabolism were two outstanding pathways enriched by the up- and down-regulated mRNAs. Thus, we speculate that the DEMs may take part in the CC process via Wnt signaling and drug metabolism.

Prognostic biomarkers for cancer have the power to change the course of a disease if they add value beyond knew prognostic factors [45]. *ULBP2*, which located on the chromosome 6, is a gene that encoding the cell surface glycoprotein [46]. A previous study shows that *ULBP2* is a novel prognostic biomarker for CC [47]. Demirkol et al. indicated that *ULBP2* was a mRNA based stage-independent prognostic marker to prognosticate CC in vivo [48]. In the analysis of pancreatic cancer, researchers have proved that a high level of soluble *ULBP2* is deemed an independent indicator for OS [49]. In this study, *ULBP2* was identified as the unique mRNA outstanding in all three datasets. Thus, we speculate that *ULBP2* may be used as the OS prognostic biomarker in CC. Furthermore, *GRP* is a regulatory molecule that has been implicated in a number of physiological and pathophysiological processes [50]. A Previous study shows that *GRP* can better predict the prognosis of patients with colorectal cancer and distant metastasis, and has good sensitivity and specificity [51]. Matkowskyj et al. confirmed that *GRP* and its receptor’s co-expression had the function of differentiation, with highest levels observed in well-differentiated CC cells [52]. Moreover, the transmembrane protein is a type of integral membrane protein that spans the entirety of the biological membrane to which it is permanently attached [53]. Based on the results of previous studies, various transmembrane proteins such as MutL Homolog 1 (*MLH1*) and Bone Marrow Stromal Cell Antigen 2 (*BST2*) are associated with the progression of CC [54, 55]. Unfortunately, there is no related study based on transmembrane protein *TMEM37* and CC. In the current study, independent DFS prognostic genes analysis showed that *GRP* and *TMEM37* were two most outstanding mRNAs, which might be used as the independent DFS prognostic genes. However, a further clinical investigation based on a large scale of sample size is still needed to confirm the thesis speculation.

## Highlights

1. *ULBP2* was a novel overall survival prognostic gene in CC.
2. *GRP* was an independent disease-free survival prognostic gene in CC.
3. *TMEM37* was an independent disease-free survival prognostic gene in CC.
4. Extracellular matrix and digestion functions were important for CC.
5. Wnt signaling and drug metabolism pathways were vital for CC.

## Abbreviations

CC: colon cancer
TCGA: The Cancer Genome Atlas
OS: overall survival
DFS: disease-free survival
